# Digitally supported shared decision-making and treat-to-target in rheumatology: a qualitative study embedded in a multicenter randomized controlled trial

**DOI:** 10.1007/s00296-022-05224-y

**Published:** 2022-10-14

**Authors:** Felix Muehlensiepen, Susann May, Katharina Hadaschik, Nicolas Vuillerme, Martin Heinze, Manuel Grahammer, Hannah Labinsky, Sebastian Boeltz, Jacqueline Detert, Jana Petersen, Gerhard Krönke, Georg Schett, Johannes Knitza

**Affiliations:** 1 Center for Health Services Research, Faculty of Health Sciences Brandenburg, Brandenburg Medical School Theodor Fontane, Rüdersdorf Bei Berlin, Germany; 2 Université Grenoble Alpes, AGEIS, Grenoble, France; 3 Institut Universitaire de France, Paris, France; 4 LabCom Telecom4Health, Orange Labs and Univ. Grenoble Alpes, CNRS, Inria, Grenoble INP-UGA, Grenoble, France; 5 Abaton GmbH, Berlin, Germany; 6 Department of Internal Medicine 3, Friedrich-Alexander-University Erlangen-Nürnberg and Universitätsklinikum Erlangen, Erlangen, Germany; 7 Deutsches Zentrum Immuntherapie (DZI), Friedrich-Alexander-University Erlangen-Nürnberg and Universitätsklinikum Erlangen, Erlangen, Germany; 8 Rheumatologisch-Immunologische Arztpraxis Templin, Templin, Germany; 9 Klinik für Rheumatologie and Immunologie, Klinikum Bad Bramstedt GmbH, Bad Bramstedt, Germany

**Keywords:** Rheumatology, ePRO, SDM, mHealth, Qualitative research, Health Services Research

## Abstract

Patient-reported outcomes (PRO) represent a cornerstone in the management of patients with rheumatoid arthritis (RA). However, PRO are currently recorded mainly on paper and only during on-site appointments. Electronic PRO (ePRO) enable continuous remote monitoring and could improve shared decision-making (SDM) and implementation of a treat-to-target (T2T) approach. This study aims to investigate patient and physician experiences, perceived drawbacks and benefits of using an ePRO web-app (ABATON RA) to digitally support SDM and T2T. A qualitative study embedded in a multicenter randomized controlled trial (RCT) consisting of interviews with RA patients and physicians that were subsequently analyzed using deductive-inductive qualitative content analysis. Between August 2021 and May 2022, interviews with ten RA patients and five physicians were completed. Three key themes emerged in the analysis: (i) App user experiences; (ii) perceived drawbacks of app-supported rheumatology care; and (iii) perceived benefits of app-supported rheumatology care. Continuous ePRO collection and a high level of standardization strained some RA patients. Certain ePRO seemed outdated and were hard to understand. Patients and physicians appreciated having an improved overview of disease activity, capturing disease flares and continuous remote monitoring. Paper- and time-saving were associated with using ePRO. Physicians feared to become too focused on ePRO data, stressed the lack of ePRO monitoring reimbursement and app interoperability. For RA patients and physicians, benefits seemed to outweigh observed drawbacks of the digitally supported SDM using ePRO. The software was easy to use and could lead to a better understanding of the individual disease course, resource allocation and treatment of rheumatoid arthritis.

**Supplementary Information:**

The online version contains supplementary material available at 10.1007/s00296-022-05224-y.

## Introduction

Rheumatoid arthritis (RA) is a chronic inflammatory disease that requires lifelong medical care. Patient-reported outcomes (PRO) represent a cornerstone in the management of RA patients. This is exemplified by the patient’s global self-assessment (PGA) of disease activity on a 0–100 scale where 100 means maximal activity, being part of the composite gold standard to evaluate disease activity, the Disease Activity Score 28 (DAS-28) [1]. Besides the PGA, a variety of RA-specific PRO are used in clinical routine [2]. Some PRO, such as the Rheumatoid Arthritis Impact of Disease score (RAID) and Rheumatoid Arthritis Disease Activity Index-Five (RADAI-5), cover different facets of disease and have validated cut-off values for low, medium and high disease activity, potentially allowing patients and clinicians to get a quick disease activity overview. Currently, disease activity is mainly evaluated during face-to-face appointments and no PROs are collected in between visits. Due to the declining number of rheumatologists [3], the recommended tight face-to-face monitoring is increasingly difficult to implement in clinical routine. Additionally, RA disease activity fluctuates, often causing disease flares in between appointments.

Electronic PRO (ePRO) enable continuous remote monitoring and could improve monitoring of disease activity by capturing otherwise overlooked changes of disease activity [4]. Shaw et al. further reported that discussion of ePRO data leads to an improved patient–provider relationship [5]. A major challenge, not limited to RA and rheumatology is poor ePRO adherence [4, 6–8]. Poor adherence can stem from a multitude of factors, including but not limited to lack of perceived benefit [9], age and high disease activity among patients [10] or lack of integration into clinical workflows and electronic health records among professionals [11, 12]. In a recent review, Wiegel et al. concluded that to optimize adherence to tele-monitoring with ePRO, mixed-method studies are needed [7]. Despite the advantages of ePRO, only a minority of rheumatologists is currently using them in clinical routine in Germany [2].

The aim of the prospective multicenter AORTA (AbatOn for RheumaToid Arthritis) trial was to investigate the benefit of using an ePRO web-app, ABATON RA, to support shared decision-making (SDM) and treat-to-target (T2T) in RA patients. The aim of this embedded qualitative study was to investigate patient and physician experiences, perceived drawbacks and benefits of using the ePRO web app, ABATON RA, to digitally support SDM and T2T.

## Materials and methods

The participants of this qualitative study were RA patients who were randomized to the intervention group (IG) of the AORTA trial and physicians in rheumatology care.

### AORTA trial

**Fig. 1:**
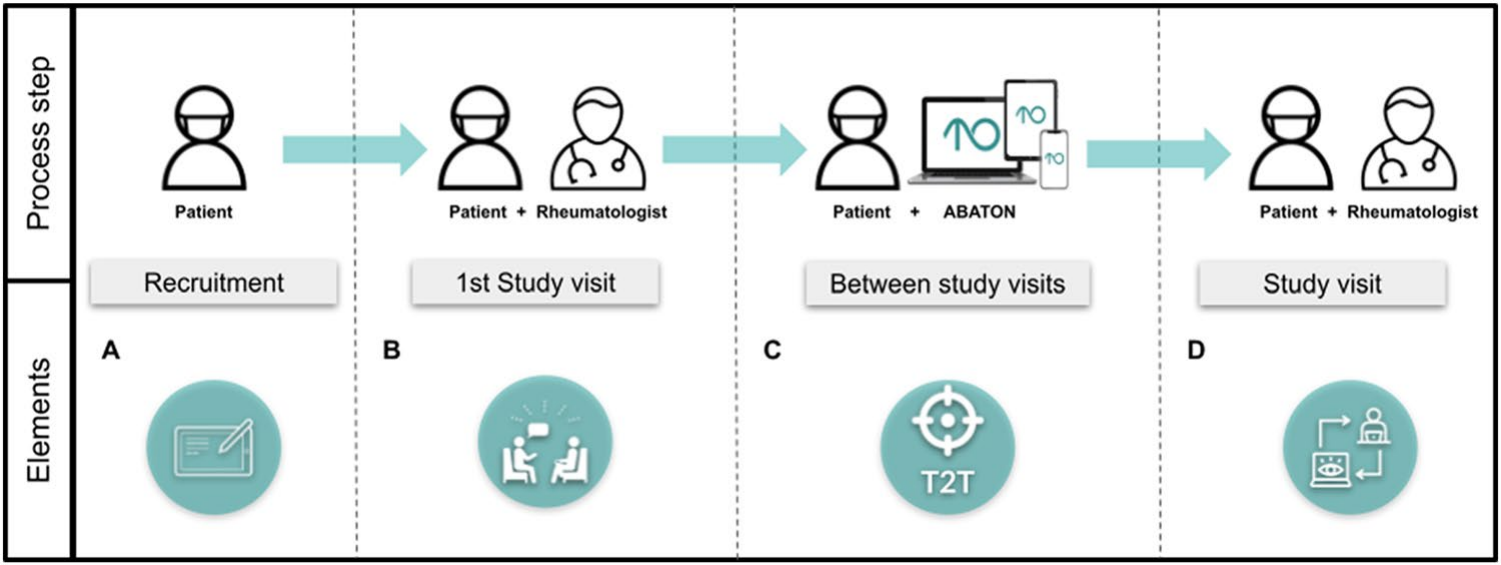
Process of digitally supported ePRO-based monitoring in the intervention group using ABATON RA. **A** Informed consent during recruitment; **B** Shared decision-making between HCP and patient to decide on (I) which Patient-Reported Outcome (PRO) to focus on e.g., Rheumatoid Arthritis Impact of Disease (RAID), (II) which target value to set e.g., 2.8 and (III) which frequency to measure e.g., fortnightly; **C** Patient tracks and self-evaluates the PRO + target. **D** Evaluation of the PRO charts and the target + all elements from (**B**)

AORTA is a three-armed, partially blinded multicenter randomized controlled trial (RCT) with four visits, each being 3 months apart. The IG patients used the web app ABATON RA to implement ePRO, SDM and T2T. At baseline, rheumatologists presented their patients 6 ePRO to choose from, including RAID [13], RADAI-5 [14], Health Assessment Questionnaire (HAQ) [15], Funktionsfragebogen Hannover (FFbH) [16], pain (0–10 numeric ratings scale) and disease activity (PGA) (0–10 numeric ratings scale) and together with the patient set a therapy goal using this ePRO for the next visit, see Fig. 1. Each week the patient is reminded to complete the selected ePRO and these data are then discussed at the next face-to-face visit with the treating rheumatologist. In the placebo group (PG), patients have access to a sham version of the app to collect two sleep ePRO, the Regensburger Insomnie Skala (RIS) [17] and the Epworth Sleepiness Scale (ESS) [18]. In the control group (CG), patients had no access to the app. Physicians had no access to ePRO results in the PG and CG and no ePRO-based SDM or T2T was carried out.

### Web-App: ABATON RA

**Fig. 2:**
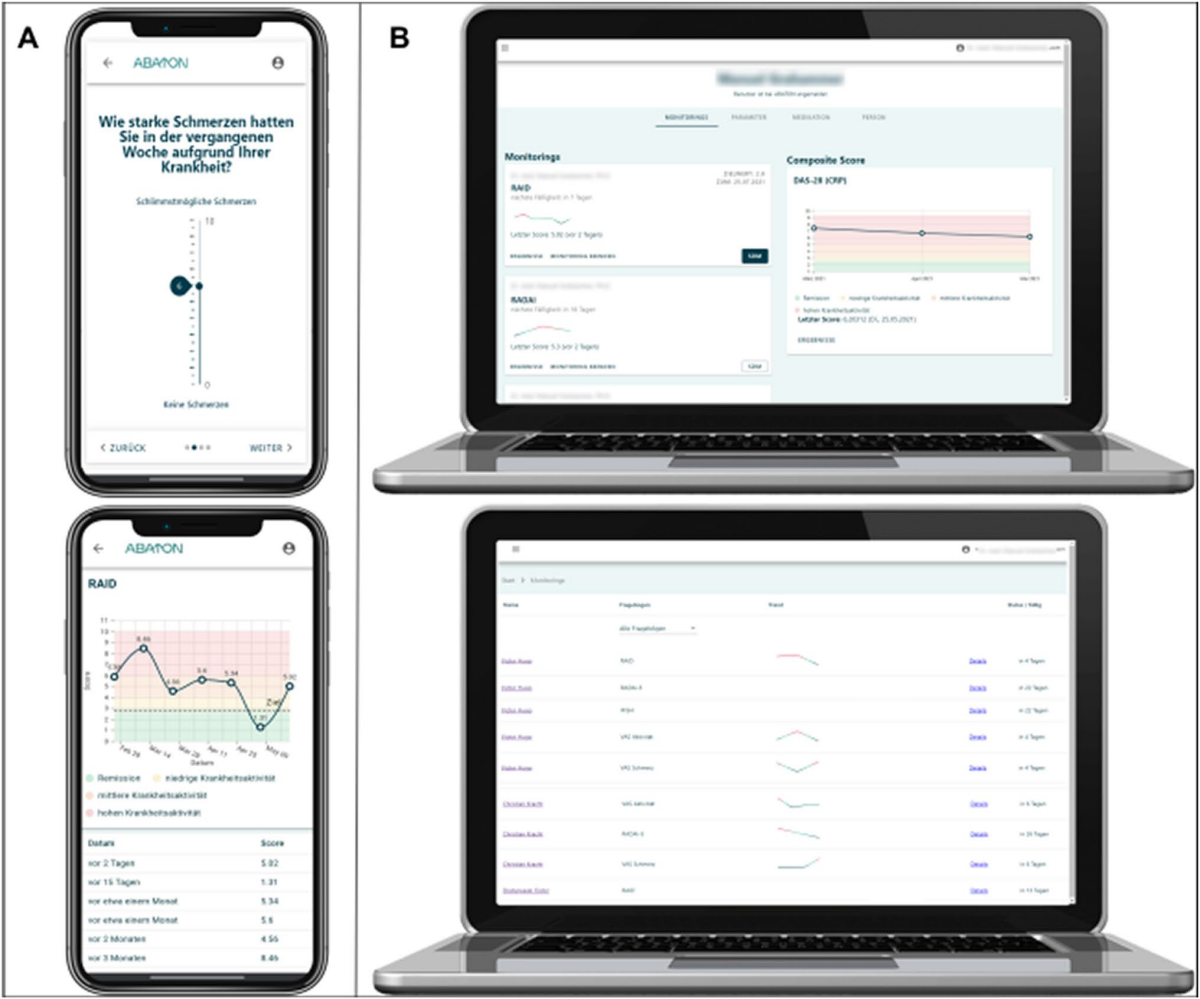
Screenshots of patient smartphone dashboard (**A**) and the physician dashboard (**B**)

The ABATON RA app is a medical device developed and maintained by ABATON GmbH (Berlin, Germany). All digital administered questionnaires, forms and monitoring instruments were pre-configured. Patients get invited by their local care team via a short messaging system (SMS) invite which contains a personalized link to set a password. Using this password and the mobile phone number patients can theoretically login to their account on any device, as ABATON RA is a web app. Patients were however instructed to use the app on their smartphones only. Patients can set if they want to be reminded of new questionnaires via push notifications or SMS. A reminder logic is implemented to remind the patients 3 consecutive days if they have not filled out the questionnaires and stop as soon as the due questionnaire is completed. Results are immediately available to the patient (Fig. 2a) and the treating healthcare team via the web-based dashboard (Fig. 2b), including graphical trends.

### Qualitative study

To explore the user experiences with the app, we conducted qualitative phone interviews with IG RA patients from one center (University Hospital Erlangen, Germany) and participating physicians from three German centers (University Hospital Erlangen, Hospital Bad-Bramstedt and Rheumatological-immunological medical practice Templin) that had used the software for at least 3 months. Participants were selected using purposive sampling [19], to include a heterogeneous sample in regard of age, sex, education and professions of the patients interviewed. Patients of the IG-group selected as potential interview participants were asked during routine appointments if they were interested in participating in a qualitative phone interview. Participants did not receive financial incentives. All patients approached agreed and provided written informed consent. The principal investigator passed the patients’ contact information to the study team for the qualitative study.

Interviews were conducted by two health services researchers (FM and SM), and one medical student (K.H.) using two analogous open-ended interview guides that were developed to elicit patients’ and physicians’ perspectives on app-supported rheumatology care. The interview guides were developed by F.M., one physician (J.K.) and one app developer (M.G.). The interview guides (Supplemental Material 1 and 2) included the following three main topics: 1. Study procedure and participants’ experiences; 2. Description of the app and its usability; 3. Impact of the app on rheumatology care. Initial exploratory questions were then specified by follow-up questions. We conducted pilot interviews to test and refine the interview guides. No revisions were necessary. Additional sociodemographic data were collected, including gender, age, diagnosis, education and occupation or medical practice. To reduce the risk of infection and lower participant burden, the interviews were conducted via telephone.

The interviews were audio-recorded and transcribed verbatim. Qualitative analysis of the interviews was performed iteratively by F.M. and S.M. based on Kuckartz’s structured qualitative content analysis [20] using MAXQDA software (Verbi GmbH). Relevant text passages from the interview material were coded according to a deductive–inductive procedure. Categories were developed based on the research questions and merged into a coding tree, which was then discussed by the members of the study team. At this stage, data collection had already been completed. The coding tree was applied to the entire interview material and partially extended with new codes, which emerged from the interview material. To ensure traceability, F.M. and S.M. independently applied the final coding tree (Supplemental Material 3) to the entire material. For the presentation of the results, representative quotes of the transcripts were selected, translated into English and included into the manuscript, while long quotes were visually set off from the main text.

## Results

### Participant characteristics

From August to December 2021, we conducted qualitative interviews with 10 RA IG patients and from February to May 2022 with five physicians, see Table 1. Mean age of interviewed patients and physicians was 51 (range 27–73) and 34 (range 28–49) years, respectively. Half of the interviewed patients were female (5/10; 50%). Patients reported diverse occupational and educational backgrounds**.** Interviews with patients lasted an average of 28 (8–77) minutes. Most physicians were assistant physicians (*n* = 4), and female (3/5; 60%)**.** Interviews lasted an average of 25 (10–47) minutes.

**Table 1:** Participant characteristics

Participant	Age	Gender	Education	Occupation
Pat 1	58	F	Middle secondary school	Secretary
Pat 2	73	M	University of applied sciences	Pensioner
Pat 3	65	F	None	Pensioner
Pat 4	41	F	Vocational training	Cleaner
Pat 5	56	M	Lower secondary school	Delivery man
Pat 6	57	F	High school	Physiotherapist
Pat 7	50	M	University of applied sciences	Manager—workshop
Pat 8	61	F	Vocational training	Pensioner
Pat 9	48	M	University of applied sciences	Educator
Pat 10	27	M	Lower secondary school	Baker
HCP 1	30	M	University degree	Assistant doctor
HCP 2	28	F	University degree	Assistant doctor
HCP 3	30	F	University degree	Assistant doctor
HCP 4	34	M	University degree	Assistant doctor
HCP 5	49	F	University degree	Private practice doctor

### Themes

The analysis followed three key themes: (i) App user experiences; (ii) perceived drawbacks of app-supported rheumatology care; and (iii) perceived benefits of app-supported rheumatology care. The results of the key themes are presented separately for patients and physicians.

#### App user experiences

##### Patient perspective

Patients described the app as easy to use. They highlighted the user interface of the app: *“I find the app brilliant, because the questions are presented beautifully. Not flashy and colorful, but really neutral.” (P8, pos. 123)*. Patients reported that they use the app primarily after being reminded that a new questionnaire is available for completion (*“when I have to fill out a questionnaire again.” (P5, pos. 18)*); or to track their disease progression *(“It's always interesting to take a look: 'How was it in March or April?'” (P2, pos. 234)*). Most patients described the app as helpful in gaining an overview of their own disease activity. Some reported that the use of the app gave them a feeling of support or security, took away fear or had a motivating effect: *“The ABATON RA app is such a hold for me, it makes me feel calmer. Because I see that [the disease] is slowing down, it’s working, [the medication] is kicking in and it's great and everything's in the green.” (P8, pos. 128).*

Yet, most patients reported opportunities for improvement: Some of the questions asked (e.g., weekly Funktionsfragebogen Hannover (FFbH) [16]) were difficult to understand or ambiguous: *“'Is there any difficulty in turning a faucet on and off?' Well, I do not know where there are still faucets nowadays that you have to turn on and off.” (P10, pos. 25).* Other patients criticized the high degree of standardization and repetition of the questionnaires, while calling for more specific or differentiated answer options, e.g., to link changes in the disease state and lifestyle changes: *“Last time the doctor told me that my score was very good in July. And I was in rehab, but he can't know that. I can't enter it anywhere. It wouldn't be bad if you could simply enter something like that as a patient.” (P6, pos. 103)*. Finally, patients proposed that users themselves should be able to determine the times at which they are reminded of questionnaires.

##### Physician perspective

Overall, the interviewed physicians described the app as well-structured and easy to use, while some mentioned initial difficulties: *“I had a few technical difficulties at the beginning. Those diminished, once you understand a little bit how it works.” (HCP 2, pos. 45)*. Physicians reported that they use the app to prepare for the consultation, after the consultation, to follow up disease status after medication changes, and most importantly during consultation:

*“I ultimately rebuild the whole consultation, usually I always ask ‘How was it last week?’. I have a certain pattern and now with ABATON RA it's completely different. You can start by saying ‘Let's take a look at the course of your illness or the last three months.’ and then look at the screen together. So just turning the screen around is something completely new that I've never done before. (…) So it's somehow easier to get into a conversation with the patient and the patients also feel better understood, because often you don't see the symptoms at the doctor's visit. And then the patient can show you: ‘Look over here, two weeks ago I felt bad and then again four weeks ago’. Thus he can also refer to it. So I think the patient also feels better understood if he can show you something, as if he then sits in front of me with a bad conscience and says ‘yes, I'm currently doing well, but three weeks ago I was doing badly, but I can't really show you anything now, like that’.” (HCP 1, pos. 26–27).*

The participating physicians consider the app as an additional aid for most patients to gain better overview of their disease and increase treatment adherence and motivation; while other patients lack motivation to use the app: *“And yes, then the patients simply do not fill out these questionnaires and then mention technical difficulties as an excuse. But then it works again during the consultation. So technical difficulties are often used as a bit of an excuse for not using it.” (HCP 1, pos. 37)*. This might also be due to the high level of standardization and repetition of the questions: *“Many patients complain that it's always the same, but that's exactly the whole purpose of the app, isn't it? And that works super reliably.” (HCP 2, pos. 23).* Physicians reported that the use of the app ultimately re-defines the roles in the relationship between patients and their treating medical staff, as those can follow and audit the medical documentation:

*“In other words, patients do take a look at what you document in the app. And when it comes to medication, for example, you really do have access to the same data. In standard care, patients have no access to our medical documentation. And [with the app] we really do share the same data. So it's just very unusual, because normally you're somehow a bit untouchable. You can document whatever you want, and it's really the first time that ‘I don't just check the patient’ to see whether he's taken his medication or had any vaccinations. Often one is nevertheless in such a control function, but with the app also vice versa; whether I have also documented the whole thing cleanly.” (HCP 1, pos. 47)*

#### Perceived drawbacks of app-supported rheumatology care

##### Patient perspective

Patients also reported limitations of app-supported ePRO documentation. For example, due to the high degree of standardization, the app was perceived as too superficial to encourage self-reflection: *“But as it is right now, the app is rather for regular communication with the doctor, so that he knows how I am, not bad, but for self-reflection it is not enough for me.” (P6, pos. 89)*. Therefore, the patient perceived the benefit of the app actually only on the doctor's side: *“Yes, it doesn't change the care at all, I think. (…) The doctor looks at it. I believe that he knows everything better. And then I think it's important for the doctor and not for me. He has to explain it to me. That's how I see it.” (P6, pos. 60)*. One participant described that constant pursuit of one's own disease activity can potentially lead to negative thoughts: *“If you dwell too often on your own disease activity, it can be associated with negative thoughts. You may be more likely to get into such a negative vortex. [The app] will keep reminding you of your disease.” (P9, pos. 101)*. Another drawback reported was that the entered information might be inaccurate due to recall bias: *“But ultimately it's like this, you tend to answer from the gut: Yes, I'm fine today. One tends to remember less about how it was five or seven days ago.” (P7, pos. 35)*. While patients reported that particularly individuals with a smartphone, technical skills, high health literacy and disease knowledge would be suitable to use the app, persons who do not meet these characteristics are left out: *“If someone does not work with a smartphone, he has no idea how to do it, he needs guidance.” (P3, pos. 42)*. This also applies to patients who do not have access to technical devices: *“And there are also a lot of people who simply don't have the money. (…) Having a compatible device actually also involves a lot of money” (P6, pos. 36).*

##### Physician perspective

A central drawback reported by the interviewed physicians was to become very focused on app data and get biased before the appointment, no longer perceiving the patients and their needs holistically: *“Of course, you have to be careful not to become too much of a data junkie and then ignore everything else, right?” (HCP 5, pos. 61).* In line with the patient perspective, interviewed physicians reported that ePRO are subjective and prone to error: *“You just have to answer the questionnaires honestly. There are certainly some who are only doing it to please me and perhaps don't take the answers seriously.” (HCP 1, pos. 39)*. The interviewed physicians considered it a limitation that the app was only available for rheumatoid arthritis. Moreover, general limitations of ePRO were pointed out: *“The problem, which always exists, is that you can't really differentiate on the basis of the questions and the app, is it rheumatoid arthritis or is it fibromyalgia, which a lot of patients have.” (HCP 3, pos. 33)*. Another drawback reported was that ePRO app use is not incorporated in the remuneration system for rheumatological care in Germany: *“So it would not be feasible at the moment because, of course, if the patients don't visit because they're doing well, we can't earn any money or bill them for anything. And also this monitoring of patient input is currently not yet remunerated, i.e., only minimally.” (HCP 1, pos. 57)*. In addition, one physician reported that more and more mhealth applications are finding their way into clinical routine, hence integrating them all into everyday practice and electronic health records is a challenge:

*“That's always my nightmare. Every patient waves his smartphone because he has collected some kind of data, and then I have to compare and evaluate them all. That's my nightmare, of course. That's why I have an interest in making sure that the data is interoperable and can be easily merged, right? (...) Well, that starts with the interface definition. This must somehow be integrated into my practice management system. And then, as I said, the daily work routine is very complex. And I need the information at a glance.” (HCP 5, pos. 41).*

Finally, as mentioned by patients, physicians reported that patients expect the discussion of the entered ePRO, which may mean extra work: *“Because, of course, for patients, using the app at home sometimes feels a bit pointless, when the findings or the individual results are not discussed. In this respect, all patients who have used the system regularly actually respond to this. And they then also demand that you look into the app together and also help them in interpreting the data.” (HCP 2, pos. 57).*

#### Perceived benefits of app-supported rheumatology care

##### Patient perspective

Major benefits of app-supported rheumatology care reported by the patients are the possibility to continuously monitor their health, to receive a clear overview of the disease progression, as well as, to provide the treating medical staff with better and more comprehensive insights. Patients emphasized that they could use the app to show or even prove their disease activity, specifically deterioration of the health status, to their medical staff: *“And I mean, the doctor always immediately sees all the information that I constantly enter as well as how I've been doing in the last few weeks. That is positive, too. You can also prove that.” (P4, pos. 65)*. According to most patients, app use encourages reflection on their own disease, which is why they described the app to be helpful for other diseases and medical areas as well: Multiple sclerosis, pain management, medication management, as well as other rheumatological diseases. In addition, patients reported that using the app, they save paper and time at the rheumatology ward, which they usually need to fill out paper-based PRO-questionnaires.

##### Physician perspective

Overall, the benefits of app-supported rheumatology care reported by patients were consistent with those of interviewed physicians:

*“Frequent documentation, closer monitoring of the clinical patient, that's something on the one hand. Of course, also the agreement of a common therapy goal - by simply being able to define the patient outcome, which is also understandable for the patient. The patient then understands his starting point and perhaps also where he can reach or perhaps where he should stay. And then there is also compliance promotion, patient education involved.” (HCP 2, pos 25)*

Furthermore, physicians emphasized that the use of the app can lead to time savings in rheumatology care: *“I rather feel that it leads to a saving of time, because you talk relatively concretely about complaints, relapses that have occurred. Or it's quite clear in the ABATON RA app if everything was just fine. You don't even have to look any further. Because you can just see that in the graph. The patient kept answering these questionnaires.” (HCP 2, pos. 33)*. Thus the app might ultimately promote a more effective rheumatology care delivery: *“Well, it's more effective because I open the program before I call the patient in, I look at the data and I know before the patient comes in whether it's going well or not and whether we probably have to change the therapy or not.” (HCP 3, pos. 31)*. Physicians emphasized that the app could be used to implement need-adapted rheumatology care: *“(…) in such a way that I see mainly patients who just deteriorated. But then to be able to see them more quickly and perhaps also patients who are demonstrably doing well, who perhaps only need to be spoken to briefly by telephone or not at all and simply only once a year. So more flexible patient management.” (HCP 1, pos. 55)*. Physicians also reported that app-based continuous documentation of ePRO could be helpful in other medical domains, such as multiple sclerosis, heart failure, chronic kidney disease, diabetes, or pre- and post-operative in orthopedics.

## Discussion

In this qualitative study, we explored RA patients’ and physicians’ experiences using a new ePRO web app ABATON RA, including drawbacks, as well as benefits of app-supported rheumatology care. Overall, the results demonstrate the feasibility of digitally supported SDM and T2T, ease of use of the app and the overall dominance of observed benefits.

Users appreciated having a better overview of disease activity. Some RA patients perceived the app as supportive for their care, i.e., making disease flares but also disease improvement visible, graphically and in numbers. Similarly, physicians felt better prepared for the appointment and treatment decisions. Compared to traditional paper-based PRO, users reported the potential of time-saving and paper reduction. The high level of repetition and standardization, as well as potentially inaccurate data and difficult-to-understand PRO questions were reported as limitations. Physicians feared to become too focused on ePRO data, stressed the lack of reimbursement and interoperability. Participants stressed that some patients might be left out due to a lack of technical skills and equipment.

Collection and graphical display of disease activity has been previously identified as a main app function, desired by rheumatic patients and can support care in multiple ways [21]. Capturing of flares allows patients and physicians a more complete picture of disease activity to enable better informed treatment decisions. Incorporation of PRO results into treatment decisions is by no means clinical routine, as we could demonstrate in a previous survey, where only 23% of German rheumatologists stated to review PRO results of every patient [2]. The survey also highlighted the importance of interoperability and reimbursement to successfully implement ePRO. Qualitative results of a similar study [8] also reported gaining insight into their disease activity course as the main benefit for patients. Interestingly, in this study, patients felt less dependent on their physicians and thought that ePRO use could lead to a reduction in the number of outpatient consultations, as mentioned by *HCP1* in this study. The potential of saving resources by implementing ePRO is in line with previous studies [22–24]. The potential to safely reduce “unnecessary” visits using ePRO has been demonstrated in two RCTs [23, 24], and is increasingly being adopted into clinical routine. Necessity of physical visits was based on ePRO cut-offs, exactly the purely data-driven approach *HCP 5* feared. As ePRO are purely subjective, additional objective laboratory data could improve a data-driven monitoring approach. Previous studies showed the high interest of patients in self-collection of blood [25, 26] and a recent trial reported high-accuracy for RA-antibody and CRP levels [27]. As reported in a similar study by Zuidema et al. [28], one of our participants described the app use to be confronting and continuous ePRO documentation potentially associated with negative thought of users. The authors also recommended screening patients for ePRO eligibility, with patient characteristics being in line with our study. Furthermore, similar to *P3*, Navarro-Millán et al. reported that providing patients with social support might enhance PROs collection by helping them overcome barriers with using electronic devices and patients’ reservations about the value of these data [29].

This study has some limitations. First, all patients were recruited in a single study site, thus results might not be generalizable. Second, even though all of the physicians interviewed were practicing in rheumatology care, only one physician was a finished-trained rheumatologist; the other four physicians were completing their residency training in rheumatology. Furthermore, participating physicians did not have access to any preliminary results of the AORTA trial and did only describe their individual study experiences. The choice of ePRO and reasons for it were not discussed. Recall bias cannot be excluded, e.g., due to time difference between app use and the interview. In addition, results may be biased toward the benefits as participants agreed to participate in the study in the first hand. A major strength of this study is the open and explorative study design and the diversity of the included patients (age, sex, education and occupation).

## Conclusion

This study shows that digitally supported SDM using ePRO is perceived as beneficial and feasible by RA patients, assistant and specialist physicians, who participated in qualitative interviews. Study participants of both user groups reported that the approach trialed could improve current rheumatology care.

## Supplementary Information

Below is the link to the electronic supplementary material.

Supplementary file1 (DOCX 20 KB)

Supplementary file2 (DOCX 21 KB)

Supplementary file3 (DOCX 14 KB)

Supplementary file4 (PDF 481 KB)

## Data Availability

All data relevant to the study are included in the article or uploaded as supplementary material. For further questions regarding the reuse of data, please contact the corresponding author (F.M.).
