# Supplementary material for: Digitally supported shared decision-making and treat-to-target in rheumatology: a qualitative study embedded in a multicenter randomized controlled trial
Source: Rheumatol Int. 2022 Oct 14;43(4):695–703. doi: 10.1007/s00296-022-05224-y (PMC9995411; doi:10.1007/s00296-022-05224-y)
Supplement: Supplementary file 1 — Supplementary file1 (DOCX 20 KB) [file 296_2022_5224_MOESM1_ESM.docx]

**Supplemental Material 1.** Interview Guide - Patient

| **Guiding Questions** | **Check Aspects** |
| --- | --- |
| You have been using the [XX] app for a few weeks as part of a study.  **Please describe. How did you come to participate in the study?**  Follow-up questions:  What did you do in the study? Please describe. | Study procedure, study experience |
| **Please describe the [XX] app to me.**  Follow-up questions:  - When do you use the app?  - How long does it take you to use the app?  - How often do you use the app? (Does the app change your daily life?)  - What works well?  - Is there anything that doesn't work well?  - How do you perceive its usability?  - Did you receive an introduction / informational materials? Were these helpful?  - How did the download / installation work?  - Do you understand the app?  - Do you understand the instructions of the app?  - Do you see any risks in using the app?  - Is there anything you would change about the app?  - Is the documentation effort appropriate?  - Is there anything else that stood out to you? | App description, usage behavior, frequency of use, functionality, usability, comprehensibility, risks, potential of improvement, appropriateness |
| **Does the app [XX] have any impact on your rheumatology care? Could you please describe?**  Follow-up aspects:  - Does the app help you to get a better overview of your disease activity? If so, how?  - Does the app have any influence on your consultations with the rheumatologist?  - Do you discuss the app data during the consultation?  - Does the app influence your health status? If so, in which way?  - Do you think the app could be used in other medical domains (outside rheumatology care)? | Changes in care delivery, risks, influence on medical consultations, health status, utility in other medical domains |
