# Supplementary material for: Digitally supported shared decision-making and treat-to-target in rheumatology: a qualitative study embedded in a multicenter randomized controlled trial
Source: Rheumatol Int. 2022 Oct 14;43(4):695–703. doi: 10.1007/s00296-022-05224-y (PMC9995411; doi:10.1007/s00296-022-05224-y)
Supplement: Supplementary file 2 — Supplementary file2 (DOCX 21 KB) [file 296_2022_5224_MOESM2_ESM.docx]

**Supplemental Material 2.** Interview Guide - Physicians

| **Guiding Questions** | **Check Aspects** |
| --- | --- |
| For several weeks now, the [XX] app is being trialed as part of a pilot study in your [name organizational unit].  **Please describe. How did you come to participate in the study?**  Follow-up questions:  What did you do in the study? Please describe. | Study procedure, study experience |
| **Please describe your experiences with the [XX] app to me.**  Follow-up questions:  - What are the functions of the app in the rheumatology treatment?  - How do you integrate the app into your rheumatology care delivery? Please describe.  - When do you access the data?  - How long does it take?  - How did the download / installation work?  - Do you understand the app?  - Do you understand the instructions of the app?  - Do you see any risks in using the app?  - Is there anything you would change about the app?  - Is the documentation effort appropriate?  - What are the benefits of using the app in rheumatology care?  - What are the drawbacks of using the app in rheumatology care?  - Is there anything else that stood out to you?  - How much time do you need to spend with patients who use the app compared to patients who do not use the app? [Why is that?] | App description, functions, integration into rheumatology care / routines, usage behavior, frequency of use, usability, comprehensibility, risks, potential of improvement, appropriateness |
| **Does the app [XX] have any impact on your rheumatology care? Could you please describe?**  Follow-up aspects:  - What influence does the app have on the conversation with the patients?  - Do you refer to the data documented in the consultation? Please describe.  - Do patients also contact you outside the consultation hours to talk about the app / ask questions? If yes, what are these about?  - Does the app influence patients’ health status? If so, in which way?  - Do you think the app could be used in other domains (outside rheumatology care)? | Changes in care delivery, influence on medical consultations, health status, utility in other medical domains |
