# Supplementary material for: Digitally supported shared decision-making and treat-to-target in rheumatology: a qualitative study embedded in a multicenter randomized controlled trial
Source: Rheumatol Int. 2022 Oct 14;43(4):695–703. doi: 10.1007/s00296-022-05224-y (PMC9995411; doi:10.1007/s00296-022-05224-y)
Supplement: Supplementary file 3 — Supplementary file3 (DOCX 14 KB) [file 296_2022_5224_MOESM3_ESM.docx]

**Supplemental Material 3. Coding Tree**

| **Theme** | **Categories** | **Code (Examples)** |
| --- | --- | --- |
| App user experiences | Usability | Comprehensibility, functionality, readability, bugs, errors, compatibility, introduction, download, installation, device… |
|  | App description | Interface, layout, content, purpose, functions, appropriateness… |
|  | Usage behavior | Usage modes, duration of use, time of data entry, consultation, emotions while using the app, reflection… |
|  | Potential of improvement | Questionnaires, open questions, password, reminders… |
| Perceived drawbacks of app-supported rheumatology care | ePRO documentation | Standardization, repetition, bias prone, superficial, time consuming, no differentiation of diseases, binding nature… |
|  | Adverse effects | “Negative vortex”, focus on data (not patient)… |
|  | Exclusion potential | Low health literacy, low technical skills, not able to afford devices, other medical conditions… |
|  | Administration | Financial losses, integration into medical routines, access data management, technical support… |
| Perceived benefits of app-supported rheumatology care | Empowerment | Improved disease monitoring, common therapy goal, role allocation (patient / physician), reflection, improved communication, proof of own condition… |
|  | Time savings | Faster consultations (physicians), visits at the medical practice, access to patient data… |
|  | Need-adapted rheumatology care | Continuous monitoring, independence, flexibility, medication check in between visits, effectiveness of rheumatology care… |
|  | Other medical domains | Other rheumatological diseases, multiple sclerosis, pain management, heart failure, chronic kidney disease, diabetes, orthopedics… |
